# Histological evidence for the cardiac safety of high-dose pegylated liposomal doxorubicin in a patient with HIV-associated Kaposi sarcoma: a case report and literature review

**DOI:** 10.1186/s12879-019-4500-7

**Published:** 2019-10-15

**Authors:** Ayaka Ishihara, Shuji Hatakeyama, Jun Suzuki, Yusuke Amano, Teppei Sasahara, Masaki Toshima, Yuji Morisawa

**Affiliations:** 1 Division of Infectious Diseases, Jichi Medical University Hospital, 3311-1 Yakushiji, Shimotsuke-shi, Tochigi, 329-0498 Japan; 2 Division of General Internal Medicine, Jichi Medical University Hospital, 3311-1 Yakushiji, Shimotsuke-shi, Tochigi, 329-0498 Japan; 3 Department of Diagnostic Pathology, Jichi Medical University Hospital, 3311-1 Yakushiji, Shimotsuke-shi, Tochigi, 329-0498 Japan; 4 Department of Infection and Immunity, School of Medicine, Jichi Medical University, 3311-1 Yakushiji, Shimotsuke-shi, Tochigi, 329-0498 Japan

**Keywords:** Human immunodeficiency virus infection, Kaposi sarcoma, Kaposi sarcoma-associated herpesvirus inflammatory cytokine syndrome, Pegylated liposomal doxorubicin, Cardiac toxicity

## Abstract

**Background:**

Pegylated liposomal doxorubicin plays an important role in the treatment of patients with severe refractory human immunodeficiency virus (HIV)-associated Kaposi sarcoma (KS). High cumulative doses of conventional doxorubicin exceeding 500 mg/m^2^ are known to cause cardiac toxicity. However, the safe cumulative dose of pegylated liposomal doxorubicin is unclear.

**Case presentation:**

A 40-year-old Japanese man with HIV infection presented with pain, edema, and multiple skin nodules on both legs which worsened over several months. He was diagnosed with HIV-associated KS. He received long-term pegylated liposomal doxorubicin combined with antiretroviral therapy for advanced, progressive KS. The cumulative dose of pegylated liposomal doxorubicin reached 980 mg/m^2^. The patient’s left ventricular ejection fraction remained unchanged from baseline during treatment. After he died as a result of cachexia and wasting, caused by recurrent sepsis and advanced KS, an autopsy specimen of his heart revealed little or no evidence of histological cardiac damage. We also conducted a literature review focusing on histological changes of the myocardium in patients treated with a cumulative dose of pegylated liposomal doxorubicin exceeding 500 mg/m^2^.

**Conclusions:**

This case report and literature review suggest that high (> 500 mg/m^2^) cumulative doses of pegylated liposomal doxorubicin may be used without significant histological/clinical cardiac toxicity in patients with HIV-associated KS.

## Background

Kaposi sarcoma (KS) is a major life-threatening complication associated with human immunodeficiency virus (HIV) infection. KS is caused by human herpesvirus 8 (HHV-8)/KS-associated herpesvirus (KSHV) infection, which also causes primary effusion lymphoma and HIV-associated multicentric Castleman disease. The incidence of HIV-associated KS has decreased and prognosis has improved dramatically since the development and introduction of antiretroviral therapy (ART). It has been reported that 35% of all patients with HIV-associated KS have an advanced form of the disease, such as visceral involvement, tumor-associated edema or ulceration, or extensive oral KS [1].

Systemic chemotherapy combined with ART is recommended for advanced KS, and pegylated liposomal doxorubicin is the first-line drug used for this purpose. However, there is an emerging problem of patients who do not respond to the combination of chemotherapy and ART. A questionnaire surveillance study of HIV-associated KS in Japan showed that approximately 10% of patients had KS that was refractory to pegylated liposomal doxorubicin therapy combined with ART [2]. In patients with advanced HIV-associated KS, published studies have shown response rates to pegylated liposomal doxorubicin therapy of 46–76% [3, 4]. For these patients with refractory KS, decisions need to be made regarding a change in therapy to paclitaxel, a second-line agent, or continuation of pegylated liposomal doxorubicin for an extended period [5].

The main concern regarding long-term use of doxorubicin is dose-dependent cardiac toxicity. Based on a combined index of signs, symptoms, and decline in left ventricular ejection fraction (LVEF), cardiac toxicity is estimated to occur in 3–20% patients receiving cumulative doxorubicin doses of 300–500 mg/m^2^. The risk of developing congestive heart failure (CHF) increases dramatically with cumulative doses of doxorubicin > 400 mg/m^2^ [6]. Therefore, a pegylated liposomal formulation of doxorubicin was developed, which poses significantly lower risk of cardiotoxicity than conventional doxorubicin [7]. However, similar to conventional doxorubicin, a total cumulative dose > 500 mg/m^2^ is not recommended because of the risk of developing CHF [8]. However, there is little evidence to support the discontinuation of pegylated liposomal doxorubicin therapy for patients with refractory HIV-associated KS when the cumulative dose exceeds 500 mg/m^2^.

Here, we present a case of severe, refractory HIV-associated KS treated with a high cumulative dose of pegylated liposomal doxorubicin after showing no response to second-line therapy with paclitaxel. The patient eventually died as a result of wasting and cachexia caused by recurrent sepsis and advanced KS, after receiving a cumulative dose of 980 mg/m^2^ of pegylated liposomal doxorubicin, and had no histological evidence of myocardial damage on autopsy. We also review the literature on other patients treated with cumulative doses of ≥500 mg/m^2^, and who received myocardial biopsy. We acknowledge that we have reported the present case as a clinical image demonstrating ^18^F-fluorodeoxyglucose-positron emission tomography and gallium-67 scintigraphy findings of KS lesions in the lower extremities [9].

## Case presentation

A 40-year-old Japanese man, who had had sex with men, presented with pain, edema, and multiple skin nodules on both legs which had worsened over several months. Although he had been diagnosed with HIV infection 20 years previously, he had not sought medical treatment. On admission, he was afebrile. His legs had hardened with numerous black nodules on dark skin. Multiple lymph nodes were palpable, including cervical, submandibular, submental, supraclavicular, and axillary lymph nodes. Breath sounds in the left lung were decreased. A chest X-ray showed bilateral pleural effusion. His CD4 T-lymphocyte count was 170 cells/μL and his HIV-1 RNA load was 68,000 copies/mL. His serum C-reactive protein level was 3.1 mg/dL and he had an HHV-8 DNA load of 6500 copies/10^6^ leukocytes in whole blood samples.

A cytological analysis of pleural effusion revealed no atypical lymphocytes suggestive of primary effusion lymphoma. Biopsy specimens of the skin nodules and submental lymph node revealed KS, identified by the presence of whorls of spindle-shaped cells and positive immunostaining for HHV-8 and D2–40. There was no clinical or pathological evidence suggestive of multicentric Castleman disease. The patient was diagnosed with HIV-associated KS with a tumor Stage 1, immune system Stage 1, and systemic illness Stage 1, according to the AIDS Clinical Trials Group Oncology Committee staging criteria [10]. He met the criteria for KS-associated herpesvirus inflammatory cytokine syndrome (KICS). Two weeks after starting ART (raltegravir and tenofovir disoproxil fumarate/emtricitabine), chemotherapy with pegylated liposomal doxorubicin at a dose of 20 mg/m^2^ every 2 weeks was initiated for KS. His LVEF before initiation of chemotherapy was 60%. After 16 courses of pegylated liposomal doxorubicin, the cumulative dose was 320 mg/m^2^. His pleural effusion had decreased, and leg edema and skin nodules had gradually improved. His LVEF was monitored and did not decrease during chemotherapy.

**Fig. 1:**
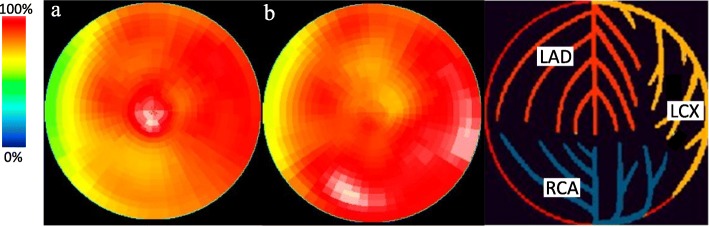
Images of ^123^I-BMIPP myocardial scintigraphy. ^123^I-β-methyl-P-iodophenyl-pentadecanoic acid (BMIPP) cardiac scintigraphy obtained after 42 courses of pegylated liposomal doxorubicin showed no reduction of ^123^I- BMIPP uptake. (**a**) Early phase (30 min after intravenous injection); (**b**) Delayed phase (4 h after intravenous injection)

In order to avoid cardiac toxicity associated with long-term use of pegylated liposomal doxorubicin, we switched his therapy to paclitaxel (100 mg/m^2^ every 2 weeks). However, his right pleural effusion increased rapidly after two courses of paclitaxel. Repeated cytology of pleural fluid revealed no atypical lymphocytes. As an echocardiogram indicated that his LVEF had not changed significantly from baseline (62% from 60%), we switched back to pegylated liposomal doxorubicin treatment. At this point, his right pleural effusion began to gradually decrease again. His skin nodules became less marked, but he developed recurrent blisters on his thighs and knees, which required application of topical antiseptics such as silver sulfadiazine cream. He did not experience any palmar-planter erythrodysthesia, thrombocytopenia, or severe neutropenia during the treatment. Cardiac scintigraphy using ^123^I-β-methyl-P-iodophenyl-pentadecanoic acid (BMIPP), performed after 42 courses of pegylated liposomal doxorubicin, showed no myocardial metabolic defect (Fig. 1).

**Fig. 2:**
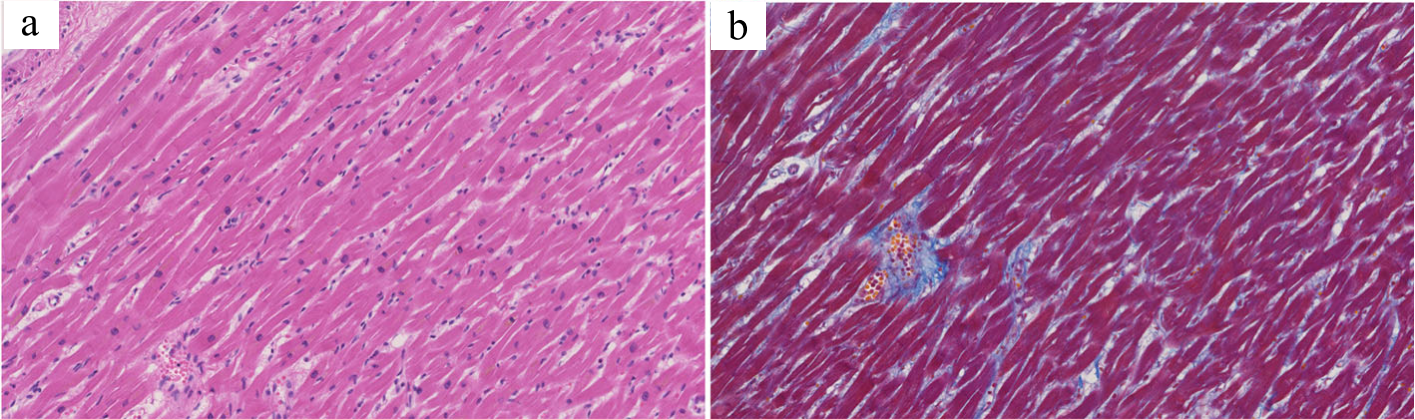
Myocardial histopathology of the autopsy specimen. (**a**) Preserved myofibrils or myocytes with little inflammatory cell infiltration were observed in hematoxylin and eosin stained tissue (original magnification × 20); (**b**) No increase in collagen between myocytes observed on azan stained tissue (original magnification × 20)

He underwent a total of 49 courses of pegylated liposomal doxorubicin, with a total cumulative dose of 980 mg/m^2^. After the 49 courses he developed septic osteoarthritis of his right hip, and pegylated liposomal doxorubicin was discontinued. He subsequently developed new skin nodules appeared on his neck, and his systemic edema progressively worsened. He developed recurrent sepsis caused by cellulitis of the lower extremities via non-intact skin, as a result of the reappearance of the KS lesions. Two months after discontinuation of pegylated liposomal doxorubicin, he died as a result of wasting and cachexia caused by recurrent sepsis and advanced KS. At the time of his death, his CD4 T-lymphocyte count was 165 cells/μL and his HIV load was undetectable. An autopsy was performed, and it revealed systemic invasion of KS, particularly in the bilateral lung and pleura, soft tissue of the pelvis, and skin in the groin area. There were no pathological findings of other malignancies, including lymphoma or multicentric Castleman disease. The adipose tissue and skeletal muscle of the whole body were extremely atrophic, characteristic of cachexia. Histological examination of his heart (Fig. 2) showed preservation of myofibrils and myocytes, with little inflammatory cell infiltration, corresponding to 0.5 points on the Billingham scale; a widely used endomyocardial biopsy score for grading anthracycline-induced myocardial damage (Appendix). This finding indicated that he had not experienced histological cardiotoxicity as a result of the 980 mg/m^2^ cumulative dose of pegylated liposomal doxorubicin that he had received.

## Discussion and conclusions

The most reliable indicator of cardiac damage is histological change, and the Billingham scale (Appendix) has a high sensitivity for detecting early cardiac damage [11]. Myocardial biopsies from patients who develop conventional doxorubicin-induced CHF typically have a Billingham score of 3.0 [12, 13]. In this patient, there was little evidence of histological or clinical cardiac toxicity, even after receiving a total cumulative dose of 980 mg/m^2^ pegylated liposomal doxorubicin for HIV-associated KS.

Liposome encapsulation reduces the cardiac toxicity of doxorubicin while preserving its antitumor effect [14]. In a trial for metastatic breast carcinoma, non-pegylated liposomal doxorubicin showed lesser cardiac toxicity than that of conventional doxorubicin; CHF occurred in 2 and 8% of patients treated with non-pegylated liposomal doxorubicin and conventional doxorubicin, respectively [15]. Pegylation of liposomal doxorubicin reduces uptake by reticuloendothelial system (liver or spleen), and therefore maintains high blood concentrations of the drug [16]. In a study of 509 women with metastatic breast cancer, cardiac toxicity (defined by declining LVEF) during treatment and follow up was less frequently associated with pegylated liposomal doxorubicin than with conventional doxorubicin; cardiotoxicity developed in 4% versus 19% of cases, cardiotoxicity with signs and symptoms of CHF in 0% versus 4% of cases, and cardiotoxicity without signs and symptoms of CHF in 4% versus 15% of cases, respectively [7]. Several trials and case reports of breast and gynecological cancer have shown that patients treated with pegylated liposomal doxorubicin showed no signs or symptoms of CHF, even when they received a cumulative dose of pegylated liposomal doxorubicin > 1000 mg/m^2^ [17–21]. In contrast, 0–5% of patients experienced a reduction in LVEF of ≥20% relative to baseline [17–20]. There are currently limited data regarding the cardiac safety/toxicity of high cumulative doses of pegylated liposomal doxorubicin in patients with HIV-associated KS. In one study of 52 individuals with HIV-associated KS, none of the participants developed clinical heart failure after a mean cumulative dose of 456 mg/m^2^ pegylated liposomal doxorubicin [22].

Radiotherapy that involves incidental exposure of the heart is one of the risk factors for developing doxorubicin-induced cardiotoxicity. A previous study showed that the cumulative anthracycline dose and average radiation dose to the heart were significant independent factors associated with cardiac failure [23].

It has been proposed that LVEF may not accurately indicate cardiotoxicity in adult patients receiving high cumulative doses of pegylated liposomal doxorubicin [24]. We therefore conducted a literature review that focused on histological changes of the myocardium in patients treated with ≥500 mg/m^2^ of pegylated liposomal doxorubicin, in order to assess the evidence that high cumulative doses of this drug formulation can be administered without cardiac toxicity. As shown in Table 1, we identified 14 patients reported to have received ≥500 mg/m^2^ pegylated liposomal doxorubicin, who underwent myocardial biopsy for HIV-associated KS (7 patients), breast cancer (5 patients), or ovarian cancer (2 patients). The median cumulative pegylated liposomal doxorubicin dose among these patients was 708 mg/m^2^ (range, 500–1485 mg/m^2^). The Billingham scores of all patients were under 1.5, indicating that no patient had significant histological myocardial damage.

**Table 1:** Endomyocardial biopsy scores of patients treated with high cumulative doses of pegylated liposomal doxorubicin

Patient	Age	Sex	Underlying disease	Prior doxorubicin dose (mg/m^2^)	Cumulative PLD dose (mg/m^2^)	Billingham score	Reference
1	Unknown	M	KS	0	500	1	[25]
2	Unknown	M	KS	0	541	0	[25]
3	35	F	Breast cancer^a^	180	564	0.5	[26]
4	Unknown	M	KS	0	578	0	[25]
5	Unknown	M	KS	0	610	0.5	[25]
6	54	F	Breast cancer^a^	0	675	0.5	[27]
7	47	F	Breast cancer^a^	0	685	0	[26]
8	66	F	Ovarian cancer	0	730	0	[26]
9	Unknown	M	KS	0	780	0	[25]
10	Unknown	M	KS	0	801	0.5	[25]
11	Unknown	M	KS	0	860	0	[25]
12	36	F	Ovarian cancer	375	952	0	[26]
13	52	F	Breast cancer^a^	360	1320	1.5	[26]
14	49	F	Breast cancer	0	1485	1	[26]

Abbreviations: *M* Male, *F* Female, *KS* Kaposi sarcoma, *PLD* Pegylated liposomal doxorubicin

^a^Patients 3, 6, 7, and 13 received prior radiotherapy to the breast

KICS is a recently described syndrome that is characterized by severe inflammatory cytokine dysregulation attributed to lytic activation of HHV-8/KSHV. Proposed diagnostic criteria for KICS require that all of the following conditions be met: (1) At least two symptoms, laboratory, or radiographic abnormalities (including fever, fatigue, edema, cachexia, respiratory symptoms, gastrointestinal disturbance, arthralgia and myalgia, altered mental state, neuropathy, cytopenia, hypoalbuminemia, hyponatremia, lymphadenopathy, hepatosplenomegaly, and effusions of the body cavities); (2) evidence of systemic inflammation (elevated C-reactive protein); (3) elevated KSHV viral load in peripheral blood mononuclear cells; and (4) exclusion of multicentric Castleman disease [28]. Since KICS may be associated with severe inflammatory symptoms, a poor clinical response to treatment, and high mortality, it is important to recognize that individuals with severe, refractory, or recurrent KS that does not respond to standard treatment, need to be screened for KICS in addition to primary effusion lymphoma and multicentric Castleman disease.

In conclusion, both our patient and previous reports suggest that a cumulative dose of pegylated liposomal doxorubicin exceeding 500 mg/m^2^ is not necessarily associated with significant clinical/histological cardiac damage. Because alternative therapies are limited, long-term and multi-course pegylated liposomal doxorubicin treatment is occasionally required for refractory HIV-associated KS. Further studies are warranted to identify the cumulative dose of pegylated liposomal doxorubicin that is the threshold for clinical/histological cardiac toxicity in patients with HIV-associated KS.

## Data Availability

All data containing relevant information to support these findings are included in the manuscript.

## Appendix

Morphologic grades for anthracycline-induced myocardial toxicity according to the Billingham scoring system [11, 12]

**Table:**

Grade	Morphology
0	Normal myocardial ultrastructural morphology
0.5	Not completely normal but no evidence of anthracycline-specific damage
1	Isolated myocytes affected and/or early myofibrillar loss; damage to < 5% of all cells
1.5	Changes similar to those in grade 1 except damage involves 6–15% of all cells
2	Clusters of myocytes affected by microfibrillar loss and/or vacuolization, with damage 16–25% of all cells
2.5	Many myocytes (26–35% of all cells) affected by vacuolization and/or myofibrillar loss
3	Severe, diffuse myocyte damage (>35% of all cells)

## Abbreviations

ART: Antiretroviral therapy
BMIPP: ^123^I-β-methyl-P-iodophenyl-pentadecanoic acid
CHF: Congestive heart failure
HHV-8: Human herpesvirus 8
HIV: Human immunodeficiency virus
KICS: Kaposi sarcoma herpesvirus-associated inflammatory cytokine syndrome
KS: Kaposi sarcoma
KSHV: Kaposi sarcoma-associated herpesvirus
LVEF: Left ventricular ejection fraction
